# No Evidence of Dengue Virus Circulation in Rural Gabon

**DOI:** 10.3201/eid1708.110153

**Published:** 2011-08

**Authors:** Xavier Pourrut, Dieudonné Nkoghé, Jean-Paul Gonzalez, Eric Leroy

**Affiliations:** Author affiliations: Institut de Recherche pour le Développement, Montpellier, France (X. Pourrut, E. Leroy);; Centre International de Recherches Médicales, Franceville, Gabon (X. Pourrut, D. Nkoghé, J.-P. Gonzalez, E. Leroy);; Ministère de la Santé Publique, Libreville, Gabon (D. Nkoghé)

**Keywords:** dengue virus, viruses, Gabon, serology, circulation, humans, monkeys, letter

**To the Editor:** Dengue virus (DENV) is a mosquito-borne RNA virus belonging to the family *Flaviviridae*. It is composed of 4 closely related serotypes designated DENV-1–4. There are 2 transmission cycles for this virus. The endemic/epidemic cycle involves humans and the mosquito species *Aedes aegypti* and *Ae*. *albopictus*. The zoonotic or sylvatic cycle involves monkeys and sylvatic *Aedes* spp. mosquitoes (*1*).

Despite occasionally severe clinical forms, human dengue usually consists of a self-limited febrile disease often associated with asthenia, headache, rash, arthralgia, and myalgia. DENV is widely distributed throughout Asia, the Pacific, Central and South America, the Middle East, and Africa (*2**,**3*). In Africa, most DENV outbreaks have been reported in the eastern regions, and episodic cases have occurred in western regions. However, few data are available for central regions.

In Gabon, concurrent of transmission of DENV and chikungunya virus was documented in 2007 during a large outbreak of dengue (*4*). This outbreak affected Libreville and major cities in northwestern Gabon and was caused by DENV-2. DENV isolates were closely related to strains from Asia, suggesting that the outbreak resulted from recent introduction of the virus. Epidemic DENV strains are constantly moving from one region to another, and local DENV transmission from sylvatic to urban areas has been documented in some countries in Africa (*5**,**6*).

To examine possible circulation of DENV in Gabon, we tested the following for antibodies against dengue: villagers living in rural areas, pet monkeys in the same areas, and wild monkeys killed in forests for bushmeat. A total of 4,341 persons and 186 pet monkeys were sampled during July 2005–May 2008 in 220 randomly selected villages, which represented 10.3% of all villages in Gabon. Fifty wild monkeys were also sampled during October 2009–August 2010 in different regions of Gabon (Table).

**Table:** Prevalence of IgG and IgM against dengue virus in humans and nonhuman primates, Gabon*

Province	No. positive/no. tested (% positive)							
	Humans			Pet monkeys†			Wild monkeys‡	
	IgG	IgM		IgG	IgM		IgG	IgM
Estuaire	5/286 (1.7)	1/95 (1.1)		0/13 (0)	0/13 (0)		0/1 (0)	0/1 (0)
Haut-Ogooué	1/364 (0.3)	0/80 (0)		0/8 (0)	0/8 (0)		0/1 (0)	0/1 (0)
Moyen-Ogooué	3/558 (0.5)	1/113 (0.9)		0/10 (0)	0/10 (0)		0/7 (0)	0/7 (0)
Ngounié	0/146 (0)	0/60 (0)		0/51(0)	0/51 (0)		0/5 (0)	0/5 (0)
Nyanga	1/774 (0.1)	0/151 (0)		0/33 (0)	0/33 (0)		0/2 (0)	0/2 (0)
Ogooué Ivindo	5/499 (1)	0/105 (0)		0/31(0)	0/31 (0)		0/3 (0)	0/3 (0)
Ogooué-Lolo	0/416 (0)	0/90 (0)		0/11 (0)	0/11 (0)		0/3 (0)	0/3 (0)
Ogooué-Maritime	2/197 (1)	1/72 (1.4)		0	0		0/18 (0)	0/18 (0)
Woleu-Ntem	3/831 (0.4)	2/163 (1.2)		0/29 (0)	0/29 (0)		0/10 (0)	0/10 (0)
Total	20/4,341 (0.5)	5/930 (0.5)		0/186 (0)	0/186 (0)		0/50 (0)	0/50 (0)

*Ig, immunoglobulin. †*Cercopithecus cephus, C. nictitans, C. neglectus, C. pogonias, C. solatus, Cercocebus torquatus, Gorilla gorilla, Lophocebus albigena, Mandrillus sphinx, Miopithecus ogoouensis*, and *Pan troglodytes* species. ‡*Cercopithecus cephus, C. nictitans, C. neglectus, C. pogonias, Cercocebus torquatus, Mandrillus sphinx*, and *Perodictitus potto* species.

DENV-specific immunoglobulin (Ig) G and IgM were detected by using capture ELISA kits (Panbio; Brisbane, Queensland, Australia) (*7*) according the manufacturer’s instructions. All samples were tested with an IgG assay, which was designed to detect high antibody titers usually associated with secondary dengue infection. All IgG-positive human samples, 910 randomly selected IgG-negative human samples, and all monkey samples were also tested by using an IgM capture ELISA.

In humans, overall prevalences of DENV-specific IgG and IgM were 0.5% (20/4341) and 0.5% (5/930), respectively, and only 1 of 20 IgG-positive samples was IgM positive. No IgG or IgM against DENV was detected in pet or wild monkeys. Although the IgG capture ELISA did not enable us to detect low and medium titers, absence of IgM and IgG against DENV in pet and bushmeat monkeys indicates a lack of recent or secondary infections. In humans, the low prevalences of IgM and IgG against DENV suggest that DENV does not circulate actively in rural Gabon, although the 2 assays do not detect late primary DENV infection.

Although these ELISAs have been extensively validated (diagnostic sensitivity of 98.6% and specificity of 99.4%) (*7*), some of our samples may have shown false-positive results because IgG cross-reactivity between flaviviruses can occur (*8*). The rare serum specimen positive for IgG against DENV may also indicate a low level of DENV circulation in rural Gabon, or may be related to travel to and from neighboring countries. After the outbreak in Gabon, DENV surveillance with active detection of febrile human cases is needed to determine whether this virus will become endemic to this area.
